# Cost-effectiveness of early intervention in psychosis in low- and middle-income countries: economic evaluation from São Paulo, Brazil

**DOI:** 10.1017/S2045796024000222

**Published:** 2024-04-05

**Authors:** D. Aceituno, D. Razzouk, H. Jin, M. Pennington, A. Gadelha, R. Bressan, C. Noto, N. Crossley, M. Prina, for the ANDES Network

**Affiliations:** 1 Department of Psychiatry, Pontificia Universidad Católica de Chile, Santiago, Chile; 2 King’s Health Economics, Health Service and Population Research Department, Institute of Psychiatry, Psychology and Neuroscience, King’s College London, David Goldberg Centre, London, UK; 3 Mental Health Service, Complejo Asistencial Dr. Sotero del Rio, Puente Alto, Chile; 4 Centre of Mental Health Economics, Department of Psychiatry, Universidade Federal de São Paulo, São Paulo, Brazil; 5 Schizophrenia Program (PROESQ), Department of Psychiatry, Federal University of São Paulo, São Paulo, Brazil; 6 Interdisciplinary Laboratory in Clinical Neuroscience (LiNC), Department of Psychiatry, Federal University of São Paulo, São Paulo, Brazil; 7 Health Service and Population Research Department, Institute of Psychiatry, Psychology and Neuroscience, King’s College London, London, UK; 8 Population Health Sciences Institute, Newcastle University, Newcastle, UK

**Keywords:** Brazil, cost-effectiveness analysis, early intervention, economic evaluation, low- and middle-income countries, psychosis

## Abstract

**Aims:**

The effectiveness and cost-effectiveness of early intervention for psychosis (EIP) services are well established in high-income countries but not in low- and middle-income countries (LMICs). Despite the scarcity of local evidence, several EIP services have been implemented in LMICs. Local evaluations are warranted before adopting speciality models of care in LMICs. We aimed to estimate the cost-effectiveness of implementing EIP services in Brazil.

**Methods:**

A model-based economic evaluation of EIP services was conducted from the Brazilian healthcare system perspective. A Markov model was developed using a cohort study conducted in São Paulo. Cost data were retrieved from local sources. The outcome of interest was the incremental cost-effectiveness ratio (ICER) measured as the incremental costs over the incremental quality-adjusted life-years (QALYs). Sensitivity analyses were performed to test the robustness of the results.

**Results:**

The study included 357 participants (38% female), with a mean (SD) age of 26 (7.38) years. According to the model, implementing EIP services in Brazil would result in a mean incremental cost of 4,478 Brazilian reals (R$) and a mean incremental benefit of 0.29 QALYs. The resulting ICER of R$ 15,495 (US dollar [USD] 7,640 adjusted for purchase power parity [PPP]) per QALY can be considered cost-effective at a willingness-to-pay threshold of 1 Gross domestic product (GDP) per capita (R$ 18,254; USD 9,000 PPP adjusted). The model results were robust to sensitivity analyses.

**Conclusions:**

This study supports the economic advantages of implementing EIP services in Brazil. Although cultural adaptations are required, these data suggest EIP services might be cost-effective even in less-resourced countries.

## Introduction

### EIP in the global context

Early intervention in psychosis (EIP) services provide phase-specific specialised treatment to people experiencing or at high risk of developing psychosis (Fusar-Poli *et al.*, 2017; NHS England, 2016). The model of care is usually of a standalone service, with a reduced patient-to-staff ratio to enable more intensive care, better engagement, assertive outreach and explicit coordination with other levels of care (Correll *et al.*, 2018). The effectiveness and cost-effectiveness of EIP services have been consistently demonstrated across different health systems but mostly in high-income countries (Aceituno *et al.*, 2019; Correll *et al.*, 2018).

According to the Global Burden of Disease study, about 21 million people live with psychosis globally. Most of these people live in low- and middle-income countries (LMICs), where the treatment gap can be as high as 90% (Lilford *et al.*, 2020; Morgan *et al.*, 2023). Integrated EIP services offer the possibility of reducing the enduring burden associated with psychosis (Farooq, 2013; Singh *et al.*, 2023).

However, implementing EIP services in LMICs faces the ethical dilemma of adopting specialised interventions when essential services are lacking. Additional challenges, such as inadequate funding, lack of mental health policies, shortage of workforce, inadequate training and stigma toward people with mental illness, hinder mental health service development in less-resourced settings (Saxena *et al.*, 2007). Thus, before importing foreign models of care, thorough evaluations and local adaptations are warranted in order to ensure they meet people’s needs.

### EIP in Latin America

In Latin America, EIP services have been implemented in several cities but mostly aside research centres (Aceituno *et al.*, 2020a). Specifically in Brazil (the fifth-largest country in the world), EIP has proved to be feasible and acceptable in some cities, but these initiatives have not been scaled-up to the State or Federal level.

Currently, most people with psychotic disorders in Brazil receive care at the secondary level at psychosocial community centres or *Centros de Atenção Psicossocial* (CAPS) (Amaral *et al.*, 2018; Becker and Razzouk, 2018). CAPS are mental health facilities that offer outpatient care or partial hospitalisation to individuals who have been diagnosed with persistent and severe mental illnesses, regardless of their diagnosis. Multidisciplinary teams provide care according to clinical guidelines, covering a catchment area of at least 70,000 residents. However, treatment is predominantly focused on pharmacological interventions, the staff-client ratio is usually high, outreach of patients is rarely conducted and psychosocial interventions are less frequently implemented (Marchionatti *et al.*, 2023). Consequently, data from the Brazilian National Health Service (*Sistema Unico de Saúde* [SUS]) suggest that 56.2% of people with first-episode psychosis (FEP) do not receive adequate treatment (Matos *et al.*, 2015).

In this context, integrated EIP services might be an option to improve outcomes in a usually neglected population. Considering recent epidemiological studies, over 1.6 million people with psychosis live in Brazil (Del-Ben *et al.*, 2019).

However, despite the enthusiasm shown by early adopters, no information about the effectiveness or cost-effectiveness of EIP services has been published. As resources are limited, the real cost of implementing a new service is not only the service budget itself but rather the value of the benefits that could be generated if investments were made elsewhere, which is known as the opportunity cost (Drummond *et al.*, 2015). In low-resourced countries such as Brazil, it is arguably more important to make these decisions as systematic and accountable as possible.

To fill this evidence gap, our aim was to generate evidence about the cost-effectiveness of implementing EIP services in Brazil to inform local decision-making, as well as to enrich the broader discussion about implementing EIP services in less-resourced countries.

## Methods

### Study design and comparators

A model-based economic evaluation comparing EIP services against CAPS was conducted from the Brazilian healthcare system perspective. We followed the recently updated Consolidated Health Economic Evaluation Reporting Standards guidelines (Husereau *et al.*, 2022) and the Brazilian Health Technology Assessment methodological guidelines (Ministério da Saúde, 2014). We adopted a cost-utility analysis approach. Thus, EIP services and CAPS were compared in terms of their costs (in monetary terms) and effects (in quality-adjusted life-years [QALYs]). Both costs and effects were discounted at 3.0%.

### Participants and data collection

Participants were individuals 16–40 years old with FEP. They were part of the GAPi (Grupo de Apoio às Psicoses Iniciais) cohort study, a large project of the Interdisciplinary Laboratory in Clinical Neuroscience, Escola Paulista de Medicina, Universidade Federal de São Paulo (EPM/UNIFESP) aimed to study genetic and neuroimaging profiles of people at their early stages of psychosis. The GAPi cohort has been previously characterised in detail (Cavalcante *et al.*, 2019). To briefly summarise, the inclusion criteria involved antipsychotic-naive individuals with FEP who were assessed at a university-affiliated psychiatric unit in São Paulo. Participants had to be between the ages of 16 and 40, have an FEP and have no prior history of antipsychotic treatment. Individuals with psychotic symptoms due to a general medical condition, intellectual disability or acute intoxication were excluded from the study.

Participants received multidisciplinary treatment by the EPM/UNIFESP’s EIP service according to current consensus statements (Bertolote and McGorry, 2005). The EIP service provides a 3-year package of care consisting of medications (second-generation antipsychotics within the lower therapeutic range and clozapine to patients with treatment-resistant psychosis), family interventions (systemic oriented interventions with family psychoeducation), patient psychoeducation and weekly sessions of psychological therapy. An employment support group offers vocational intervention to resume work or education. The EIP service is located in a catchment area of 12.3 million inhabitants, and people with suspected psychosis are referred from primary care and emergency services.

Participants were evaluated at baseline, 3 and 12 months of follow-up. The diagnosis was assessed by the Structured Clinical Interview for Diagnostic and Statistical Manual of Mental Disorders, 4th ed, revised text (DSM-IV-TR) (American Psychiatric Association, 2000). Symptomatology was assessed using the Positive and Negative Syndrome Scale (PANSS) (Kay *et al.*, 1987), Calgary Depression Scale for Schizophrenia (Addington *et al.*, 1990), Young Mania Rating Scale (Young *et al.*, 1978) and the National Institute of Mental Health (NIMH) Measurement and Treatment Research to Improve Cognition in Schizophrenia (MATRICS) Consensus Battery (August *et al.*, 2012). Assessments were conducted by research assistants with certified training on standardised assessment.

The primary study used to populate this model received ethical approval from the Ethics Committee of UNIFESP. All patients aged 18 and above signed informed consent. For younger participants, patient and legal tutors’ consent was obtained.

### Economic model

Decision analytical models are mathematical frameworks that integrate different sources of evidence to represent a problem with the aim of informing decisions (Caro *et al.*, 2012). They are usually faster to develop and less expensive to use than clinical trials, with the additional advantages of including information usually excluded from trials (e.g. health-related quality of life). They also allow testing outcome extrapolation and scenario analyses. These features make models suitable as a vehicle to conduct economic evaluations in LMICs, where evidence-based decisions are needed, but research capacity is low, and the costs of generating local evidence are sometimes prohibitive.

**Figure 1.**
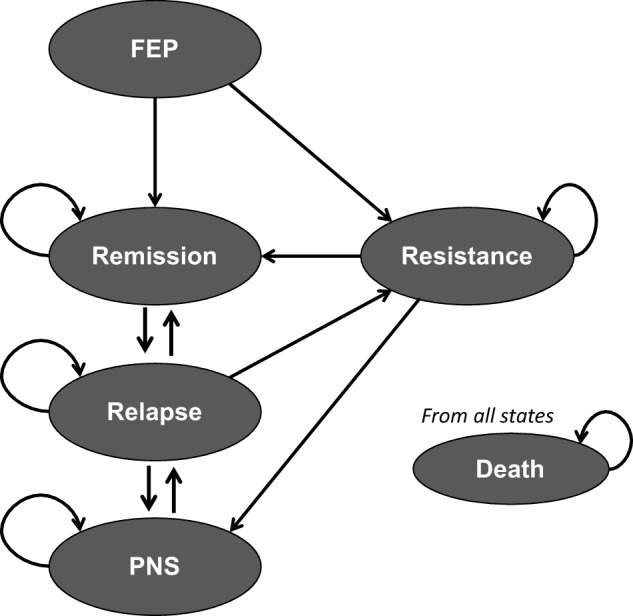
Final model conceptualisation.

We developed a cohort-level state-transition model to estimate the cost-effectiveness of EIP against CAPS based on local stakeholders’ inputs, current methodological guidelines (Caro *et al.*, 2012) and published models of psychosis (Jin *et al.*, 2020; Zhou *et al.*, 2018). State-transition models, also known as Markov models, represent the condition of interest as a series of mutually exclusive and collectively exhaustive health states. We modelled a hypothetical cohort of patients throughout six health states as presented in Fig. 1. According to Fig. 1, patients enter the model when they experience their FEP. After the FEP, patients experience varying degrees of symptom remission. Remission was defined by the PANSS according to the Remission in Schizophrenia Working Group (RSWG) criteria (Andreasen *et al.*, 2005). Those who do not recover can develop a treatment-resistant schizophrenia if they do not respond to two trials of antipsychotic medications at 600 mg equivalent of chlorpromazine, as recommended by consensus guidelines (Howes *et al.*, 2017). People achieving remission can either remain in the current state or move to the ‘Relapse’ state if they suffer a symptomatic relapse, defined as symptoms worsening requiring clinical care (Emsley *et al.*, 2013). A proportion of relapsed patients will require inpatient care to achieve symptom stability. Additionally, we included a health state to capture patients with persistent negative symptoms (Galderisi *et al.*, 2021). Finally, people in all states can move to the absorbing ‘Death’ state. The mortality rate was calculated by multiplying the age-specific mortality for the general population (derived from Brazilian life tables) and the standardised mortality ratio associated with having a psychotic illness. From the time the cohort spent on each health state, we estimated costs and QALYs associated with each interventions using a 10-year time horizon. Assumptions and simplifications of this model can be found in the supplementary materials.

FEP: first-episode psychosis, PNS: persistent negative symptoms.

### Model parameters and data sources

Following current methodological recommendations given by the National Institute for Health and Care Excellence Decision Support Unit, we conducted a rapid review on each health state included in the model (Kaltenthaler *et al.*, 2011). Search strategies and the results of each review can be found in the supplementary materials. The process to select the evidence used in the model followed a rational approach, favouring systematic reviews where possible, followed by primary studies from Brazilian sources and lastly, literature from abroad. Experts’ elicitation was used when no published evidence for a certain parameter could be found. A list with all the model’s parameters and sources is shown in the supplementary materials, Table S1.

### Measure of effectiveness

QALYs were estimated from the model using published health-state utility values (HSUVs). HSUVs represent health-related quality of life associated with a given state. The HSUVs were selected based on a systematic review of HSUVs in schizophrenia conducted by our research team and published elsewhere (Aceituno *et al.*, 2020b). Total QALYs accrued in each strategy were calculated as the overall sum of the products of HSUVs and duration of occupancy in each health state across the entire time horizon.

The effectiveness parameters of EIP services were estimated by combining individual patient-level data from the GAPi cohort with aggregate data derived from published meta-analyses (Correll *et al.*, 2018; Fusar-Poli *et al.*, 2017). Given the higher risk of bias of using observational data to estimate effectiveness, we pooled patient-level and aggregate data using the generalised Bayesian synthesis approach known as power prior (Ibrahim *et al.*, 2015; Verde and Ohmann, 2015). Briefly, in a generalised Bayesian synthesis model, the outcome of interest (e.g. odds ratio) can be modelled as the likelihood of the randomised evidence 
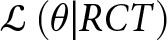
 times the likelihood of the observational evidence 
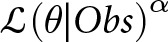
 raised to a weighting factor 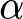. Therefore, if 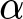 takes the value of 0, the observational evidence is completely discarded. By contrast, if 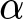 is 1 implies that the observational evidence can be considered as an additional trial.

From this approach, we estimated the mean risk ratio (RR) and 95% credible interval (CrI) of achieving remission and experiencing relapse at 1 year of follow-up in people receiving EIP services. We applied an 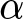 between 0 and 1 to test the impact of including observational evidence. These parameters were further plugged into the Markov model to calculate QALYs associated with the intervention.

### Service use and costs

Cost parameters were estimated by multiplying local unit costs by resource use associated with each health state. The resources were identified according to the health perspective adopted and included clinical staff, specific interventions (e.g. psychotherapy sessions), physical care and medications. A list with all the included unit costs can be found in the supplementary materials, Table S2.

CAPS and inpatient treatment unit costs were estimated from the São Paulo accounting database applying a top-down approach (Becker and Razzouk, 2018). Furthermore, we used the Brazilian Prices of Drugs Database (*Banco de Preços de Medicamentos*) to estimate the unit costs per pill in the São Paulo State (Razzouk *et al.*, 2015), as a high variation in the acquisition cost by local municipalities has been described (Razzouk, 2017). Resource use in the EIP arm was derived from published literature (Randall *et al.*, 2015), administrative data and discussion with local experts.

Costs are presented in 2018 Brazilian real (R$) and converted to US dollar (USD), adjusting for the power purchasing parity (PPP) to facilitate international comparisons. Conversion rates were obtained from the International Monetary Fund, adjusted for inflation to take into account different costing years (Shemilt *et al.*, 2010).

### Cost-effectiveness analysis

Based on the Markov model, we estimated the costs and effects of implementing EIP services against CAPS. Cost-effectiveness was expressed as the ratio of incremental costs over the incremental benefits, also known as the incremental cost-effectiveness ratio (ICER). However, unless one of the treatments is both less costly and more effective, the question of which alternative is cost-effective will depend on the value of the opportunity cost, also known as the cost-effectiveness threshold. A Brazilian cost-effectiveness threshold has not been explicitly defined (Ministério da Saúde, 2014; Soarez and Novaes, 2017). Therefore, we followed the WHO recommendations of one to three times the GDPpc as the willingness-to-pay threshold for a QALY (World Health Organization. Commission on Macroeconomics and Health *et al*., 2001).

We also used the net monetary benefit approach as a second decision rule to decide which alternative was cost-effective. In this approach, the threshold is used to transform health benefits into monetary terms which allow making comparisons on the same scale (Stinnett and Mullahy, 1998). According to the expected utility theory, the option with the highest expected net benefit is the option that maximises the chances of obtaining the preferred outcome (Claxton, 1999).

### Sensitivity analysis

In order to fully characterise the uncertainty of the parameters, as well as non-linear relationships imposed by the Markov model, we conducted probabilistic sensitivity analysis (PSA). In a PSA, the model is run multiple times using a random sample from the entire probability distribution of each parameter (Baio and Dawid, 2015). We ran the model 5,000 times using the parameterisation detailed in Table S1 in the supplementary materials.

Furthermore, a series of deterministic sensitivity analyses were conducted to assess the robustness of the model results. Firstly, we tested lower cost-effectiveness thresholds, as critics of the WHO heuristic suggest the threshold might be too high (Woods *et al.*, 2016). Secondly, we tested longer time horizons, including 20 years, 30 years and a lifetime horizon (75 years). Thirdly, we tested a scenario where the effectiveness (and the costs) of EIP interventions was assumed to last for 5 years. This is because some researchers have argued extending EIP services after the initial 3-year period (Puntis *et al.*, 2020). Fourthly, given emerging evidence of the effect of EIP on reducing patients’ mortality (Chan *et al.*, 2019), we tested that scenario as an exploratory analysis.

### Model implementation and data analysis

The model was fully implemented in the programming language R version 4.0.4 (R Core Team, 2021). Bayesian statistical models were conducted in the JAGS language version 4.3.0 (Plummer, 2018) using weakly informative priors, two Markov chain Monte Carlo chains with 50,000 iterations and a burn-in (discarded sampling) of 5,000. The source code can be found at https://github.com/david-aceituno.

## Results

### Individual-level data

Table 1 shows the baseline characteristics of the cohort data. Individual-level data were available for 357 participants. Sixty-two per cent of participants were male, with a mean (SD) age of 26 (7.38) years. Most of the participants had completed secondary education, but only a fifth had a formal job at the beginning of the study.

**Table 1.** Baseline characteristics of participants

Participants (*n*, %)	(*N* = 357)	
Sex		
Male (*n*, %)	221	62.0
Female (*n*, %)	136	38.0
Age (mean, SD)	26.0	(7.38)
Education (*n*, %)		
Illiterate	5	1.4
Primary school	44	12.3
Secondary school	137	38.3
Undergraduate	93	26.0
Postgraduate	25	7.0
Missing	53	14.8
Marital status (*n*, %)		
Single	221	61.90
Married	53	14.85
Civil partner	14	3.92
Divorced	1	0.28
Widowed	1	0.28
Missing	67	18.77
Employment status (*n*, %)		
Unemployed	110	30.8
Informal job	120	33.6
Formal job	74	20.7
Missing	53	14.8
Clinical		
PANSS positive (mean, SD)	25.62	(7.01)
PANSS negative (mean, SD)	20.71	(7.93)
PANSS total (mean, SD)	45.05	(11.74)
CDSS (mean, SD)	2.61	(4.35)

SD: standard deviation, PANSS: Positive and Negative Symptoms Scale, CDSS: Calgary Depression in Schizophrenia Scale.

### Aggregate data

We found seven randomised controlled trials (RCTs) providing aggregate-level data on the effectiveness of EIP services in achieving remission and reducing relapses (Craig *et al.*, 2004; Grawe *et al.*, 2006; Kane *et al.*, 2016; Nishida *et al.*, 2018; Petersen *et al.*, 2005; Ruggeri *et al.*, 2015; Valencia *et al.*, 2012), encompassing 1,292 participants. Other trials assessing the effectiveness of EIP services have been published (Hui *et al.*, 2015; Kuipers *et al.*, 2004), but they did not report the outcomes of interest.

A list of the included studies is shown in supplementary materials, Table S4.

### Effectiveness of EIP services

Forest plots showing the results of applying a Bayesian meta-analysis to the aggregate data are presented in the supplementary materials (Figures S2 and S3). Using a random-effects model resulted in a pooled RR of 1.41 (95% CrI: 0.98–2.03) of achieving remission in people receiving EIP services. When the outcome assessed was a relapse, the aggregate evidence suggested that people with FEP receiving EIP services had a RR of relapse of 0.66 (95% CrI: 0.33–1.02).

**Figure 2.**
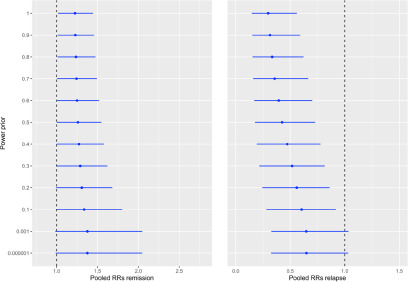
Pooled risk ratios at different levels of including observational evidence.

The effect of including the Brazilian cohort data is presented in Fig. 2. In the case of the remission parameter, including observational evidence reduced the uncertainty in the pooled estimate, with practically no change in the pooled mean estimate of remission. For instance, under the scenario of including the observational data as an additional trial (patient-level data weight of 1), the pooled RR of achieving remission for people receiving EIP was 1.22 (95% CrI: 1.01–1.44). Meanwhile, when the observational evidence was downweighed 50%, the RR changed slightly to 1.26 (95% CrI: 1.00–1.54).

Forest plots showing the effect of incorporating observational evidence within a meta-analysis of aggregate data. The *y*-axis shows increasing weighting of the observational evidence from bottom to top, as the weighting factor is exponential. The *x*-axis represents the estimated effect size (remission at the left and relapse at the right) in the risk ratio (RR) scale. The blue point-and-range lines represent pooled effect sizes at different weighting of the observational evidence.

With regard to the risk of relapse, the effect of including the observational evidence was more pronounced. When the cohort data were considered as an additional trial, the pooled RR in the EIP group fell to 0.31 (95% CrI: 0.14–0.56). The RR increased to 0.43 (95% CrI: 0.17–0.72) when the patient-level data were downweighed 50%. When the observational evidence was excluded, the pooled RR returned to 0.66 (95% CrI: 0.33–1.02).

### Economic modelling

The results of the base case economic analysis are shown in Table 2. According to this analysis, the implementation of EIP services in Brazil resulted in a mean incremental cost of R$ 4,478 and a mean incremental benefit of 0.29 QALYs. The resulting ICER of R$ 15,495 (USD 7,640 adjusted for PPP) per QALY can be considered cost-effective at a willingness-to-pay threshold of 1 GDP per capita (R$ 18,254 or USD 9,000 PPP adjusted).

**Table 2.** Results of base case analysis comparing early intervention for psychosis services against psychosocial community centres

Strategy	Mean costs (R$)	Mean effects (QALYs)	Incr. costs (R$)	Incr. effects (QALYs)	ICER
CAPS	144,278.8	5.89	NA	NA	NA
EIP	148,757.2	6.18	4,478	0.29	15,495

CAPS: Centros de Atenção Psicossocial, EIP: early intervention in psychosis, R$: Brazilian real, QALYs: quality-adjusted life-years, ICER: incremental cost-effectiveness ratio, NA: not applicable.

**Figure 3.**
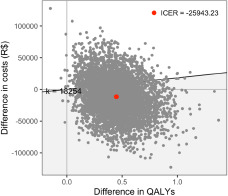
Cost-effectiveness plane.

Additionally, we plotted the simulations conducted in the PSA in the cost-effectiveness plane (Fig. 3). As shown in Fig. 3, most of the simulations fell in the southeast (65.3%) and northeast (34.4%) quadrant. Based on these simulations, the mean ICER of EIP services was estimated to be R$ −25,943 (USD −12,792 PPP adjusted) per QALY.

The figure shows the simulations of the PSA. The *x*-axis represents the difference between EIP services and CAPS in terms of QALYs. The *y*-axis represents the difference between EIP services and CAPS in terms of costs (R$).PSA: probabilistic sensitivity analysis, QALYs: quality-adjusted life-years, EIP: early intervention in psychosis, CAPS: Centros de Atenção Psicossocial, R$: Brazilian real.

Applying the net monetary benefit approach, at 1 GDPpc (R$ 18,254) of willingness to pay, EIP had a 74.3% probability of being cost-effective. When the cost-effectiveness threshold was raised to 3 GDPpc (R$ 54,762), the probability of EIP being cost-effective increased to 85.4%. Figure S4 shows the probability of EIP being cost-effective at different thresholds of willingness-to-pay.

Using remission rate as the outcome of interest, the model estimated that 58.6% of people receiving EIP would achieve RSWG-criteria clinical remission at 1 year, which is within meta-analytic estimates (Lally *et al.*, 2017). According to the model, the ICER of achieving remission at 1 year would be of R$ 2,032 (USD 1,002) per remission achieved.

### Sensitivity analysis

Assuming the intervention has an effect beyond the first 3 years increased the probability of EIP being cost-effective. The ICER was reduced to R$ −51.6 per QALY, compared to the base case of R$ 15,495 per QALY. In other words, extending EIP services would be cost-saving.

Including the effect of EIP services on patients’ mortality (Chan *et al.*, 2019) had a slight change in the base case analysis, with higher incremental benefits (0.33 QALYs) but higher costs (incremental costs R$ 149,612). As a result, the ICER increased slightly to R$ 16,151 per QALY. When the model was run using a lifetime horizon, the ICER decreased to R$ 9,629 per QALY.

## Discussion

According to this model-based economic evaluation, EIP services might be considered cost-effective compared with CAPS from the Brazilian National Health System perspective. Our analyses suggest that EIP services are cost-effective at a willingness-to-pay threshold of 1–3 GDP per capita. Furthermore, the mean ICER calculated from the PSA indicates that EIP services could potentially be cost saving. Our results were robust to conducted sensitivity analyses, and differences between deterministic and stochastic approaches can be explained by non-linearities imposed by the Markov model. Similar results were obtained when a clinical outcome (remission rate) was considered. To the extent of our knowledge, this is the first cost-effectiveness analysis of an EIP service conducted in Latin America or any other LMIC.

### Comparison with previous literature

The results of this study are consistent with published literature showing the economic advantages of implementing EIP services (Aceituno *et al.*, 2019). Previous economic evaluations of EIP services, however, have been mostly based on trials conducted in high-income countries. For instance, McCrone *et al*. (2010) found that the Lambeth Early Onset service had a 92% probability of being more cost-effective than community mental health teams in London. Similarly, using data from the Danish OPUS trial, Hastrup *et al*. (2013) found a 96.5% likelihood of EIP being cost-effective at a willingness-to-pay threshold of €2,000 per unit of Global Assessment of Functioning (GAF) improvement. Similar results can be found in cohort studies from Australia, Canada, Ireland, Italy and Sweden (Behan *et al.*, 2019; Cocchi *et al.*, 2011; Cullberg *et al.*, 2006; Mihalopoulos *et al.*, 2009).

The primary outcome of interest in most of the studies has been clinical measures. Arguably, the QALY is a better measure of patient benefit, as it integrates the impact of disease on morbidity and mortality. Furthermore, QALY provides a common metric to compare different treatments and different conditions to facilitate decision-making.

Two previous trial-based economic evaluations have used QALYs as the measure of benefit. Zhang *et al.* (2014) evaluated the cost-effectiveness of a package of psychosocial interventions and medications for people with early psychosis in China. They found that the combined package was cost-effective compared to standard care with an ICER of US$1,819 per QALY gained (the common threshold accepted in China is US$5,100 per QALY gained). Similarly, Rosenheck *et al.* (2016) evaluated the cost-effectiveness of EIP services in the Recovery After an Initial Schizophrenia Episode Early Treatment Program (RAISE-ETP) trial in the US. They found that EIP had a 90% probability of being cost-effective compared to standard care but at a threshold of US$210,000 per QALY. The cost-effectiveness threshold has been largely debated in the US, ranging from US$100,000 per QALY to US$264,000 per QALY (Braithwaite *et al*., 2008).

Comparisons with other model-based cost-effectiveness analyses of early psychosis are problematic, as some models have focused on specific interventions such as liaison with primary care to improve referrals and cognitive behavioral therapy (CBT) for people at risk of psychosis (Perez *et al.*, 2015; Wijnen *et al.*, 2020).

### Strengths and limitations

This study has several strengths. Firstly, using a Bayesian approach allows the inclusion of information from different sources in a rational and transparent manner. Hierarchical models have the advantage of borrowing information from other studies, while the power prior method leverages the usefulness of the cohort data, which included 357 participants with FEP. To the best of our knowledge, this is the largest study of an EIP service from a developing country.

Secondly, the modelling development process followed an iterative approach using the best available evidence and inputs from local experts. This model tried to capture relevant health states in the natural history of people with schizophrenia while at the same time reflecting the sparse data coming from LMICs. Thirdly, the model made use of different strategies to represent parameter uncertainty and tested different scenarios which might be useful for decision-makers.

However, there are several limitations to mention. Firstly, given the absence of individual-level data on resource use and periodically published unit costs, costs parameters were estimated with high uncertainty. Several costs were obtained from costing studies conducted in the same jurisdiction of São Paulo. However, there is evidence of high variation in the costs of interventions and medications across different regions in Brazil. More granular service use data in EIP services are needed to improve the estimation of cost-effectiveness of EIP in Brazil before scaling them up to other states.

Secondly, costs outside the healthcare system were not included. Although the health system is the main payer of the intervention under evaluation, it has been widely recognised that mental disorders have economic impacts beyond the healthcare system (Park *et al.*, 2016). Of particularly, relevance is the productivity loss, as the unemployment rate for people with psychosis can be as high as 90% (Evensen *et al.*, 2016). Considering that psychosis usually develops at young ages, the long-term effect on productivity can be substantial. Similarly, the economic impact of informal caregiving has been highlighted as a relevant yet usually neglected factor in the economic evaluations of mental health interventions (Krol *et al.*, 2015). Unfortunately, no information about the cost of informal care could be found for this study. Likewise, we did not include costs borne by the social care sector, education or the criminal justice system. Published literature suggests that broadening the economic perspective may increase the benefits of EIP services (Park *et al.*, 2016).

Finally, models can be useful simplifications, but their utility is limited by the quality of the input data. We applied recognised statistical models to limit bias in the cohort analysis. However, using observational data always carries a higher risk of biased estimates compared to RCTs.

### Implications for policy and future research

Based on the available data and the model results, EIP services appear to be cost-effective in Brazil. From a health policy viewpoint, implementing EIP services would result in more efficient use of resources. However, the implementation of EIP services in Brazil faces several challenges. According to the latest WHO Mental Health Atlas, Brazil spends only 1.6% of the health budget on mental health (World Health Organization, 2021). Although similar to other LMICs, such figure is below international recommendations.

Although implementing EIP services appears to be a rational use of resources, the country may first need to invest in other mental health policies, such as improving better mental healthcare at a primary care level (2013). According to Thornicroft and Tansella (Thornicroft and Tansella, 2013), EIP services could be considered when lower levels of the mental health system are fully implemented.

Additionally, other sources of evidence must be considered before implementing nationwide policies. For example, EIP services have been proved to be feasible and acceptable in cities such as São Paulo, Rio de Janeiro and Ribeirao Preto. However, Brazil is a heterogeneous and culturally diverse country, whereby local adaptations are warranted. In this sense, the inclusion of service users is crucial to promote an adequate adaptation. This is an area to foster in Latin America.

Furthermore, there are specific contingencies in Brazil whose current policy might challenge the implementation of EIP services. First, austerity measures were introduced in 2016 (Constitutional Amendment 95), which imposed limits on the growth of public expenditure until 2036. According to Atun *et al*. (2015) and Castro *et al*. (2019), such policy threatens further expansion and sustainability of the SUS with adverse consequences on people’s health. Second, Brazil has been one of the worst-hit countries by the COVID-19 pandemic. With more than 600,000 deaths and almost 23 million cases (https://ourworldindata.org/coronavirus/country/brazil), the pandemic has also revealed large disparities across geographical areas and ethnic groups (Martins-Filho *et al.*, 2021). Emerging evidence suggests that in the aftermath of the pandemic, Brazilians’ mental health was considerable damaged (Goularte *et al.*, 2021) and the availability of services severely disrupted (Armitage, 2021). It is probably unsurprising that people with psychosis are left behind in this global crisis. Hence, EIP services might play a crucial role in protecting this vulnerable group, as highlighted by international recommendations (Jauhar *et al.*, 2021).

## Supporting information

Aceituno et al. supplementary material Aceituno et al. supplementary material

## Data Availability

The authors declare that all data supporting the findings of this study are available within the article and its supplementary information file.

## Supplementary material

The supplementary material for this article can be found at https://doi.org/10.1017/S2045796024000222.
