# Supplementary material for: Cost-effectiveness of early intervention in psychosis in low- and middle-income countries: economic evaluation from São Paulo, Brazil
Source: Epidemiol Psychiatr Sci. 2024 Apr 5;33:e21. doi: 10.1017/S2045796024000222 (PMC11022262; doi:10.1017/S2045796024000222)
Supplement: Aceituno et al. supplementary material [file S2045796024000222sup001.docx]

Supplementary materials

Cost-effectiveness early intervention in psychosis in low- and middle-income countries: economic evaluation from São Paulo,Brazil

[Supplementary materials 1](#_Toc149924240)

[1. Model development 2](#_Toc149924241)

[1.1 Assumptions and simplifications 2](#_Toc149924242)

[2. Rapid reviews for model health states 4](#_Toc149924243)

[2.1 Remission in psychosis 4](#_Toc149924244)

[2.2 Relapse in psychosis 7](#_Toc149924245)

[2.3 Treatment resistant schizophrenia 10](#_Toc149924246)

[2.4 Persistent negative symptoms 13](#_Toc149924247)

[2.5 Mortality in schizophrenia 15](#_Toc149924248)

[3. Unit costs 19](#_Toc149924249)

[4. Model parameters 21](#_Toc149924250)

[5. List of randomised trials included in the remission and relapse meta-analyses. 26](#_Toc149924251)

[6. Forest plots 27](#_Toc149924252)

[7. Cost-effectiveness acceptability curves 29](#_Toc149924253)

[References 30](#_Toc149924254)

## Model development

### Assumptions and simplifications

Assumptions and simplifications are essential to any model (Robinson 2008, Kaltenthaler et al. 2011, Roberts et al. 2012). Assumptions have been defined as ways of incorporating uncertainties and beliefs about the real world into the model. Simplifications, on the other hand, are ways of reducing the model complexity by eliminating parts that are deemed less relevant or accessory to answer the question at hand (Robinson 2008).

Although assumptions and simplifications are considered necessary when developing a model, they can also introduce bias and limitations. Current guidelines suggest transparency by detailing all assumptions and simplifications imposed to the model (Kaltenthaler et al. 2011, Roberts et al. 2012). In this way, external reviewers and decision makers can gauge how close an assumption or simplification can be compared to existent knowledge or data.

A list of the model assumptions and simplifications is provided in Table S1.

Table S1. Model assumptions and simplifications

| Assumptions | Simplifications |
| --- | --- |
| Both groups received comparable pharmacological interventions. | incident cases were not modelled. |
| Profile of comorbidities were similar in both groups. | CHR-P were not included. |
| Relapse and FEP had equivalent symptomatology | The clinical outcome of interest was remission. Therefore, recovery was not included. |
| Both groups had similar adherence rate to treatment. | Deaths due to natural and unnatural causes were modelled as one health state |
| Patients need to experience acute exacerbations of symptoms before developing TRS or PNS. |  |
| TRS and relapse had equivalent severity level of positive symptoms. |  |
| The HSUVs of schizophrenia patients in remission was considered similar to the HSUVs of the general population . |  |
| SMR of schizophrenia was considered the same in both arms. |  |
| CHR-P: clinical high-risk of psychosis, FEP: first-episode psychosis, TRS: treatment-resistant schizophrenia, PNS: persistent negative symptoms, HSUVs: health-state utility values, SMR: standardised mortality ratio | |

## Rapid reviews for model health states

Following the model conceptualisation we conducted a rapid review on each health state following the recommendations given by the National Institute for Health and Care Excellence (NICE) Decision Support Unit (DSU) (Kaltenthaler et al. 2011). The search strategies and results of each rapid review is presented below.

### Remission in psychosis

#### Search strategy

| 1. **Database**: Medline (Ovid ®) |
| --- |
| **Date**: August/2019 |
| **Filter**: yes: <http://libguides.sph.uth.tmc.edu/search_filters/ovid_medline_filters> |
| **Hits**: 250 |
| Search terms: |
| 1. remission.mp. 2. clinical remission.mp. 3. clinical recovery.mp. 4. recovery.mp. 5. response.mp. 6. exp Schizophrenia/ 7. psychosis.mp. or exp Psychotic Disorders/ 8. or/1-5 9. 6 or 7 10. (((comprehensive* or integrative or systematic*) adj3 (bibliographic* or review* or literature)) or (meta-analy* or metaanaly* or "research synthesis" or ((information or data) adj3 synthesis) or (data adj2 extract*))).ti,ab. or (cinahl or (cochrane adj3 trial*) or embase or medline or psyclit or (psycinfo not "psycinfo database") or pubmed or scopus or "sociological abstracts" or "web of science").ab. or ("cochrane database of systematic reviews" or evidence report technology assessment or evidence report technology assessment summary).jn. or Evidence Report: Technology Assessment*.jn. or ((review adj5 (rationale or evidence)).ti,ab. and review.pt.) or meta-analysis as topic/ or Meta-Analysis.pt. 11. 8 AND 9 AND 10 |

| 1. **Database**: Psycinfo (Ovid ®) |
| --- |
| **Date**: August/2019 |
| **Filter**: yes. <http://libguides.sph.uth.tmc.edu/search_filters/ovid_psycinfo_filters> |
| **Hits**: 584 |
| Search terms: |
| 1. remission.mp. or exp "Remission (Disorders)"/ or exp Symptom Remission/ 2. clinical remission.mp. 3. clinical recovery.mp. 4. exp "Recovery (Disorders)"/ or recovery.mp. 5. response.mp. 6. exp SCHIZOPHRENIA/ 7. psychotic disorders.mp. 8. exp PSYCHOSIS/ 9. (((comprehensive* or integrative or systematic*) adj3 (bibliographic* or review* or literature)) or (meta-analy* or metaanaly* or "research synthesis" or ((information or data) adj3 synthesis) or (data adj2 extract*))).ti,ab,id. or ((review adj5 (rationale or evidence)).ti,ab,id. and "Literature Review".md.) or (cinahl or (cochrane adj3 trial*) or embase or medline or psyclit or pubmed or scopus or "sociological abstracts" or "web of science").ab. or ("systematic review" or "meta analysis").md. 10. Or/1-5 11. Or/6-8 12. 10 AND 11 AND 9 |

| 1. **Database**: LILACS |
| --- |
| **Date**: August/2019 |
| **Filter**: no |
| **Hits**: 96 |
| Search terms: |
| 1. r(tw:(schizophrenia or psychosis or "psychotic disorders")) 2. (tw:(remission or "clinical remission" or "symptoms remission" or recovery)) 3. 1 and 2 |

#### Other sources of information

| **Source** | **Hits** |
| --- | --- |
| Grey literature | Not searched |

#### Search results

| Total hits databases | 930 |
| --- | --- |
| Total hits other sources | 0 |
| Total unduplicated abstracts screened | 786 |
| Full texts reviewed | 31 |
| Records included in the model | 2 |

### Relapse in psychosis

#### Search strategy

| 1. **Database**: Medline (Ovid ®) |
| --- |
| **Date**: May/2019 |
| **Filter**: no |
| **Hits**:984 |
| Search terms: |
| 1. Relapse.ti,ab,kf 2. exp Recurrence/ 3. exp Schizophrenia/ 4. psychosis.ti,ab,kf or exp Psychotic Disorders/ 5. 1 or 2 6. 3 or 4 7. 5 and 6   Limit: reviews |

| 1. **Database**: Psycinfo (Ovid ®) |
| --- |
| **Date**: May/2019 |
| **Filter**: no |
| **Hits**: 490 |
| Search terms: |
| 1. exp SCHIZOPHRENIA/ 2. psychotic disorders.ti,ab,id 3. exp PSYCHOSIS/ 4. exp "RELAPSE (DISORDERS)"/ 5. recurrence.ab,ti,id 6. 1 or 2 or 3 7. 4 or 5 8. 6 and 7   Limits: reviews |

| 1. **Database**: LILACS |
| --- |
| **Date**: May/2019 |
| **Filter**: no |
| **Hits**: 74 |
| Search terms: |
| 1. tw:Relapse 2. tw:Recurrence 3. tw:Schizophrenia 4. tw:psychosis 5. tw:Psychotic Disorders 6. 1 or 2 7. 3 or 4 or 5 8. 6 and 7 |

#### Other sources of information

| **Source** | **Hits** |
| --- | --- |
| Grey literature | Not searched |

#### Search results

| Total hits databases | 1548 |
| --- | --- |
| Total hits other sources | 0 |
| Total unduplicated abstracts screened | 1449 |
| Full texts reviewed | 29 |
| Records included in the model | 2 |

### Treatment resistant schizophrenia

#### Search strategy

| 1. **Database**: Medline (Ovid ®) |
| --- |
| **Date**: August/2019 |
| **Filter**: yes <http://libguides.sph.uth.tmc.edu/search_filters/ovid_medline_filters> |
| **Hits**: 592 |
| Search terms: |
| 1. treatment resistance.mp 2. Drug Resistance/ 3. non-response.mp. 4. resistant.mp. 5. 1 or 2 or 3 or 4 6. exp Schizophrenia/ 7. psychosis.mp. or exp Psychotic Disorders/ 8. 6 or 7 9. 5 AND 8 10. Epidemiologic Studies/ 11. cohort studies/ or longitudinal studies/ or follow-up studies/ or prospective studies/ or retrospective studies/ or cohort.ti,ab. or longitudinal.ti,ab. or prospective.ti,ab. or retrospective.ti,ab. 12. Prevalence/ or prevalence.ti,ab,kw. 13. Incidence/ or incidence.ti,ab,kw.      1. 10 or 11 or 12 or 13 2. 9 AND 14 |

| 1. **Database**: Psycinfo (Ovid ®) |
| --- |
| **Date**: August/2019 |
| **Filter**: yes. <http://libguides.sph.uth.tmc.edu/search_filters/ovid_psycinfo_filters> |
| **Hits**: 407 |
| Search terms: |
| 1. treatment resistance.mp 2. drug resistance.mp 3. non-response.mp. 4. resistant.mp. 5. 1 or 2 or 3 or 4 6. exp SCHIZOPHRENIA/ 7. psychotic disorders.mp. 8. exp PSYCHOSIS/ 9. 6 or 7 or 8 10. 5 AND 9 11. (cohort or longitudinal or prospective or retrospective).ti,ab,id. or longitudinal study.md. or prospective study.md. or retrospective study.md. 12. Incidence/ or incidence.ti,ab,id. 13. 11 or 12 14. 10 AND 13 |

| 1. **Database**: LILACS |
| --- |
| **Date**: August/2019 |
| **Filter**: no |
| **Hits**: 7 |
| Search terms: |
| 1. Treatment resistance 2. Non-response 3. schizophrenia 4. psychotic disorders 5. 1 or 2 6. 3 or 4 7. 6 and 6 |

#### Other sources of information

| **Source** | **Hits** |
| --- | --- |
| Grey literature | Not searched |

#### Search results

| Total hits databases | 1006 |
| --- | --- |
| Total hits other sources | 0 |
| Total unduplicated abstracts screened | 768 |
| Full texts reviewed | 11 |
| Records included in the model | 1 |

### Persistent negative symptoms

#### Search strategy

| 1. **Database**: Medline (Ovid ®) |
| --- |
| **Date**: August/2019 |
| **Filter**: no |
| **Hits**: 339 |
| Search terms: |
| 1. Persistent negative symptoms.mp 2. Deficit syndrome.mp 3. exp Schizophrenia/ 4. psychosis.mp. or exp Psychotic Disorders/ 5. residual schizophrenia.mp 6. 1 or 2 7. 3 or 4 or 5 8. 6 or 7 |

| 1. **Database**: Psycinfo (Ovid ®) |
| --- |
| **Date**: January/2019 |
| **Filter**: yes. Modified from Arber, 2017 |
| **Hits**: 417 |
| Search terms: |
| 1. Persistent negative symptoms.mp 2. Deficit syndrome.mp 3. exp SCHIZOPHRENIA/ 4. psychotic disorders.mp. 5. exp PSYCHOSIS/ 6. 1 or 2 7. 3 or 4 or 5 8. 6 and 7 |

| 1. **Database**: LILACS |
| --- |
| **Date**: August/2019 |
| **Filter**: no |
| **Hits**: 4 |
| Search terms: |
| 1. Persistent negative symptoms.mp 2. Deficit syndrome.mp 3. Schizophrenia 4. psychotic disorders 5. 1 or 2 6. 3 or 4 7. 5 AND 6 |

#### Other sources of information

| **Source** | **Hits** |
| --- | --- |
| Grey literature | Not searched |

#### Search results

| Total hits databases | 760 |
| --- | --- |
| Total hits other sources | 0 |
| Total unduplicated abstracts screened | 503 |
| Full texts reviewed | 31 |
| Records included in the model | 2 |

### Mortality in schizophrenia

#### Search strategy

| 1. **Database**: Medline (Ovid ®) |
| --- |
| **Date**: August/2019 |
| **Filter**: yes <http://libguides.sph.uth.tmc.edu/search_filters/ovid_medline_filters> |
| **Hits**:333 |
| Search terms: |
| 1. exp Mortality/ 2. exp Life expectancy/ 3. life-years lost 4. exp Mortality, Premature/ 5. exp Schizophrenia/ 6. psychosis.mp. or exp Psychotic Disorders/ 7. 1 or 2 or 3 or 4 8. 5 or 6 9. cohort studies/ or longitudinal studies/ or follow-up studies/ or prospective studies/ or retrospective studies/ or cohort.ti,ab. or longitudinal.ti,ab. or prospective.ti,ab. or retrospective.ti,ab. 10. (((comprehensive* or integrative or systematic*) adj3 (bibliographic* or review* or literature)) or (meta-analy* or metaanaly* or "research synthesis" or ((information or data) adj3 synthesis) or (data adj2 extract*))).ti,ab. or (cinahl or (cochrane adj3 trial*) or embase or medline or psyclit or (psycinfo not "psycinfo database") or pubmed or scopus or "sociological abstracts" or "web of science").ab. or ("cochrane database of systematic reviews" or evidence report technology assessment or evidence report technology assessment summary).jn. or Evidence Report: Technology Assessment*.jn. or ((review adj5 (rationale or evidence)).ti,ab. and review.pt.) or meta-analysis as topic/ or Meta-Analysis.pt. 11. 9 or 10 12. 7 AND 8 AND 11 |

| 1. **Database**: Psycinfo (Ovid ®) |
| --- |
| **Date**: August/2019 |
| **Filter**: yes. <http://libguides.sph.uth.tmc.edu/search_filters/ovid_psycinfo_filters> |
| **Hits**: 523 |
| Search terms: |
| 1. mortality.mp. or exp "Death and Dying"/ 2. exp Mortality Rate/ or exp Life Expectancy/ or life-years lost.mp. 3. exp SCHIZOPHRENIA/ 4. psychotic disorders.mp. 5. exp PSYCHOSIS/ 6. (cohort or longitudinal or prospective or retrospective).ti,ab,id. or longitudinal study.md. or prospective study.md. or retrospective study.md. 7. (((comprehensive* or integrative or systematic*) adj3 (bibliographic* or review* or literature)) or (meta-analy* or metaanaly* or "research synthesis" or ((information or data) adj3 synthesis) or (data adj2 extract*))).ti,ab,id. or ((review adj5 (rationale or evidence)).ti,ab,id. and "Literature Review".md.) or (cinahl or (cochrane adj3 trial*) or embase or medline or psyclit or pubmed or scopus or "sociological abstracts" or "web of science").ab. or ("systematic review" or "meta analysis").md. 8. 1 or 2 9. 3 or 4 or 5 10. 6 or 7 11. 8 AND 9 AND 10 |

| 1. **Database**: LILACS |
| --- |
| **Date**: August/2019 |
| **Filter**: no |
| **Hits**: 45 |
| Search terms: |
| 1. Mortality 2. Life expectancy 3. Premature mortality 4. schizophrenia 5. psychotic disorders 6. Psychosis 7. 1 or 2 or 3 8. 4 or 5 or 6 9. 7 AND 8 |

#### Other sources of information

| **Source** | **Hits** |
| --- | --- |
| Grey literature | Not searched |

#### Search results

| Total hits databases | 901 |
| --- | --- |
| Total hits other sources | 0 |
| Total unduplicated abstracts screened | 782 |
| Full texts reviewed | 19 |
| Records included in the model | 4 |

## Unit costs

Table S2. List of relevant resource items

| Cost component | Item | Included | Reason for exclusion |
| --- | --- | --- | --- |
| Formal healthcare sector |  |  |  |
|  |  |  |  |
| *Clinical Staff* |  |  |  |
| Psychiatrists | visits per patient | ✓ |  |
| General practitioners | visits per patient | ✓ |  |
| Occupational therapists | visits per patient | ✓ |  |
| Psychologists | visits per patient | ✓ |  |
| Social workers | visits per patient | ✓ |  |
| Mental health nurse | visits per patient | ✓ |  |
| Counsellors | visits per patient | ✕ | No data |
| Peer workers | visits per patient | ✕ | No data |
| Training | training package | ✕ | No data |
|  |  |  |  |
| *Service-level interventions* |  |  |  |
| Day care | number of days | ✓ |  |
| Inpatient care | number of days | ✓ |  |
| Residential care | number of days | ✕ | No data |
| Physical in-patient | number of days | ✓ |  |
| Emergency department visits | Visits per patient | ✓ |  |
|  |  |  |  |
| *Laboratory tests and imaging* |  |  |  |
| WBC | unit requested | ✓ |  |
| Glycemia | unit requested | ✓ |  |
| Liver function tests | unit requested | ✓ |  |
| Thyroid function tests | unit requested | ✓ |  |
| Lipid blood tests | unit requested | ✓ |  |
| Creatinine test | unit requested | ✓ |  |
| Urine drug test | unit requested | ✓ |  |
| Prolactin | unit requested | ✓ |  |
| Electroencephalogram | unit requested | ✓ |  |
| Brain scanner | unit requested | ✓ |  |
| Brain MRI | unit requested | ✓ |  |
|  |  |  |  |
| *Group interventions* |  |  |  |
| Group psychotherapy | Per session | ✓ |  |
| Family psychoeducation | Per session | ✓ |  |
| Vocational counselling | Per session | ✓ |  |
|  |  |  |  |
| *Medication* |  |  |  |
| FGA | monthly pills per patient | ✓ |  |
| SGA | monthly pills per patient | ✓ |  |
| Clozapine | monthly pills per patient | ✓ |  |
| Benzodiazepines | monthly pills per patient | ✓ |  |
| Parkinsonism management | monthly pills per patient | ✓ |  |
| Akathisia treatment | monthly pills per patient | ✓ |  |
| Metabolic syndrome management | monthly pills per patient | ✓ |  |
|  |  |  |  |
| Informal healthcare sector |  |  |  |
| Patient time | time | ✕ | Out of perspective |
| Unpaid caregiver time | time | ✕ | Out of perspective |
| Transportation costs | money | ✕ | Out of perspective |
| out-of-pockets expenses | money | ✕ | Out of perspective |
|  |  |  |  |
| Non-healthcare sectors |  |  |  |
| Productivity | Production loss | ✕ | Out of perspective |
| Consumption | money | ✕ | Out of perspective |
| Social sector | social services | ✕ | Out of perspective |
| Legal or criminal justice | crime and its consequences | ✕ | Out of perspective |
| Education | education services | ✕ | Out of perspective |
|  | | |  |

WBC: whole blood count, MRI: magnetic resonance imaging, FGA: first-generation antipsychotics, SGA: second-generation antipsychotics.

## Model parameters

The table below shows the list of all parameters included in the model with their respective sources. Mean values were used in the base case analyses, while low and high values were used in the deterministic sensitivity analysis. The distribution column shows the parameterisation applied in order to sample the parameter in the probabilistic sensitivity analysis.

Table S3 Model parameters

| Parameter | Mean value | Low value | High value | Distribution | Source |
| --- | --- | --- | --- | --- | --- |
| **Transition probabilities** | | | | | |
| Probability of remission after FEP | 0.167 | 0.054 | 0.237 | beta(𝛼 = 29.72, 𝛽 = 147.57) | (Correll et al. 2018) |
| Probability of remission after FEP (calibrated) | 0.182 | 0.161 | 0.198 | beta(𝛼 = 39.13, 𝛽 = 175.67) | Calibration |
| Probability of resistance after FEP | 0.059 | 0.041 | 0.073 | beta(𝛼 = 4.93, 𝛽 = 78.68) | (Mena et al. 2018) |
| Probability of PNS after FEP | 0.107 | 0.077 | 0.140 | beta(𝛼 = 25.59, 𝛽 = 212.91) | Experts elicitation^1^ |
| Probability of relapse after remission | 0.078 | 0.062 | 0.093 | beta(𝛼 = 62.41, 𝛽 = 735.66) | (Alvarez-Jiménez et al. 2011) |
| Probability of remission after resistance | 0.132 | 0.076 | 0.208 | beta(𝛼 = 9.34, 𝛽 = 61.02) | (Siskind et al. 2017) |
| Probability of PNS after resistance | 0.107 | 0.077 | 0.140 | beta(𝛼 = 25.59, 𝛽 = 212.91) | Assumption^2^ |
| Probability of relapse after PNS | 0.078 | 0.062 | 0.093 | beta(𝛼 = 62.41, 𝛽 = 735.66) | (Alvarez-Jiménez et al. 2011) |
| Probability of remission after PNS | 0.147 | 0.103 | 0.196 | beta(𝛼 = 22.50, 𝛽 = 130.80) | (Buchanan et al. 2012) |
| Probability of receiving Clozapine if TRS in CAPS | 0.163 | 0.119 | 0.211 | beta(𝛼 = 27.71, 𝛽 = 142.29) | (Doyle et al. 2017) |
| Probability of receiving Clozapine if TRS in EIP | 0.620 | 0.246 | 0.942 | beta(𝛼 = 2.37, 𝛽 = 1.45) | Experts elicitation |
| Probability of hospitalisation if relapse | 0.357 | 0.109 | 0.661 | beta(𝛼 = 2.50, 𝛽 = 4.45) | Experts elicitation |
| **Effectiveness** | | | | | |
| Log RR of remission in EIP | 0.231 | 0.048 | 0.411 | Normal(𝜇 = 0.231, 𝜎 = 0.110) | Bayesian MA |
| Log RR of relapse in EIP | -0.843 | -1.328 | -0.399 | Normal(𝜇 = -0.843, 𝜎 = 0.290) | Bayesian MA |
| Log RR of remission with clozapine | 0.157 | -0.211 | 0.543 | Normal(𝜇 = 0.157, 𝜎 = 0.238) | (Siskind et al. 2016) |
| Log RR of inpatient care in EIP | -0.113 | -0.204 | -0.026 | Normal(𝜇 = -0.113, 𝜎 = 0.055) | (Randall et al. 2015) |
| **Mortality** | | | | | |
| Log SMR of people with psychosis | 1.125 | 1.068 | 1.183 | Normal(𝜇 = 1.125, 𝜎 = 0.035) | (Oakley et al. 2018) |
| Log SMR of people with schizophrenia (calibrated) | 1.095 | 1.058 | 1.134 | Normal(𝜇 = 1.095, 𝜎 = 0.023) | Calibration |
| **Health utilities** | | | | | |
| Utility of FEP and relapses | 0.34 | 0.165 | 0.524 | Trunc Normal(b = 1, 𝜇 = 0.34, 𝜎 = 0.109) | (Aceituno et al. 2020) |
| Utility of remission | 0.80 | 0.745 | 0.853 | Trunc Normal(b = 1, 𝜇 = 0.80, 𝜎 = 0.033) | (Garcia-Gordillo et al. 2018) |
| Utility of resistance | 0.65 | 0.222 | 0.935 | Trunc Normal(b = 1, 𝜇 = 0.65, 𝜎 = 0.26) | (Davies et al. 2008) |
| Utility of PNS | 0.69 | 0.553 | 0.821 | Trunc Normal(b = 1, 𝜇 = 0.69, 𝜎 = 0.079) | (Aceituno et al. 2020) |
| **Costs** | | | | | |
| Per diem inpatient care costs | 371.66 | - | - | fixed | (Becker and Razzouk, 2018) |
| probability of being admitted | 0.357 | 0.109 | 0.661 | beta(𝛼 = 2.50, 𝛽 = 4.45) | Experts elicitation |
| Average length of stay | 42.98 | 30.19 | 56.04 | gamma(*k* = 31.04, 𝜃 = 1.384) | (Randall et al. 2015) |
| costs relapses CAPS^3^ | R$ 5,080 | R$2,776.86 | R$ 7,689.47 | gamma(*k* = 11.1, 𝜃 = 450.63) |  |
| costs remission CAPS | R$ 1,550 | R$901.5 | R$2,423.5 | gamma(*k* = 11.1, 𝜃 = 142.56) | (Razzouk et al. 2015, Becker & Razzouk 2018, Razzouk 2019) |
| costs resistance CAPS | R$ 8,213 | R$4,713.0 | R$12,453.2 | gamma(*k* = 11.1, 𝜃 = 732.69) | (Razzouk et al. 2015, Becker & Razzouk 2018, Razzouk 2019) |
| costs PNS CAPS | R$ 4,002 | R$2,235.5 | R$6,002.3 | gamma(*k* = 11.1, 𝜃 = 354.51) | (Razzouk et al. 2015, Becker & Razzouk 2018, Razzouk 2019) |
| costs relapses EIP^3^ | R$ 7,120 | R$3,850.1 | R$10,566.1 | gamma(*k* = 11.1, 𝜃 = 629.1) | (Razzouk et al. 2015, Becker & Razzouk 2018, Razzouk 2019) |
| costs remission EIP | R$ 1,791 | R$912.8 | R$2,580 | gamma(*k* = 11.1, 𝜃 = 147.87) | (Razzouk et al. 2015, Becker & Razzouk 2018, Razzouk 2019) |
| costs resistance EIP | R$ 10,502 | R$5,984.6 | R$16,069.8 | gamma(*k* = 11.1, 𝜃 = 938.7) | (Razzouk et al. 2015, Becker & Razzouk 2018, Razzouk 2019) |
| costs PNS EIP | R$ 5,676 | R$3,136.9 | R$8,317.9 | gamma(*k* = 11.1, 𝜃 = 499.23) | (Razzouk et al. 2015, Becker & Razzouk 2018, Razzouk 2019) |
| FEP: first-episode psychosis, PNS: persistent negative symptoms, CAPS: Centros de Atenção Psicosocial, EIP: early intervention in psychosis, RR: risk ratio, SMR: standardised mortality rate, MA: meta-analysis, 𝜇 : mean, 𝜎: standard deviation, k: shape, 𝜃: scale.  1. Parametrisation can differ from chapter 7 because of rescaling to 3-month cycle.  2. Assumed equal to probability of PNS in acute episode.  3. Does not include hospitalisation. Cost of hospitalisation was added as a probabilistic parameter in the model.  PNS defined as per the latest European Psychiatric Association guidelines (Galderisi et al. 2021)  TRS defined as per the latest Treatment Response and Resistance in Psychosis (TRRIP) consensus (Howes et al. 2017). | | | | | |

## List of randomised trials included in the remission and relapse meta-analyses.

Table S4. Studies informing aggregate data

| Study | Reference | Country | Duration (months) | Sample size |
| --- | --- | --- | --- | --- |
| LEO | (Craig et al. 2004) | UK | 18 | 144 |
| OPUS | (Petersen et al. 2005) | Denmark | 24 | 369 |
| OTP | (Grawe et al. 2006) | Norway | 24 | 50 |
| Valencia-12 | (Valencia et al. 2012) | Mexico | 12 | 88 |
| PIANO | (Ruggeri et al. 2015) | Italy | 9 | 444 |
| Valencia-6 | (Valencia et al. 2017) | Mexico | 6 | 120 |
| JCAP | (Nishida et al. 2018) | Japan | 18 | 77 |
| LEO: Lambeth Early Onset, OPUS: Specialized assertive intervention (Danish) , OTP: Optimal Treatment Project, PIANO: Psychosis early Intervention and Assessment of Needs and Outcome, JCAP: Japanese Comprehensive Approach for First-episode Psychosis. | | | | |

## Forest plots

Figure S2 Bayesian meta-analysis of remission

| 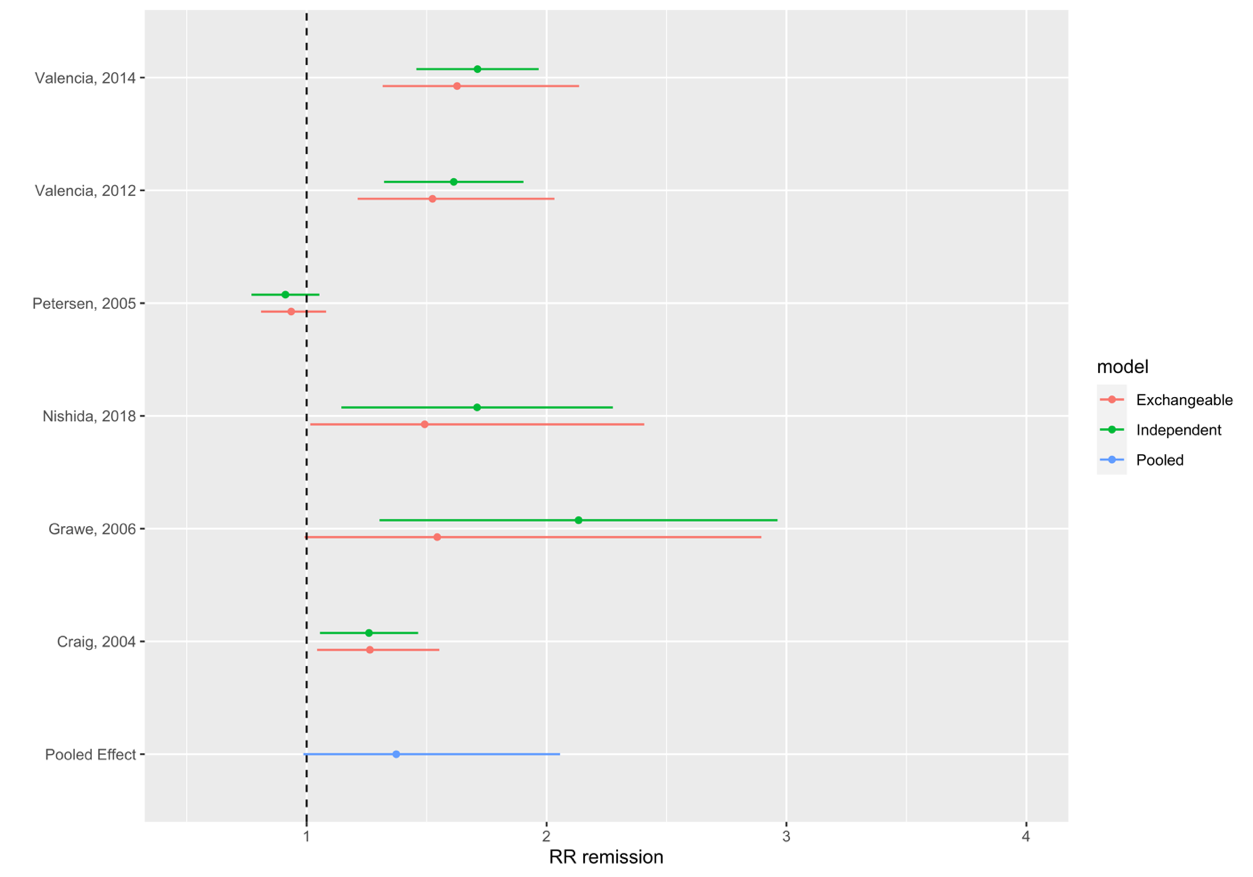 |
| --- |
| **Forest plot showing the results of a Bayesian random-effects meta-analysis of the effectiveness of EIP in terms of remission. Each green point-and-range line represents the effect size and 95% credible interval (CrI) for each study assuming they are independent. The red point-and-range lines represent the effect sizes and 95% CrI assuming exchangeability. The blue point-and-range line is the pooled effect estimate with its 95% CrI. RR: risk ratio.** |

Figure S3 Bayesian meta-analysis of relapses

| 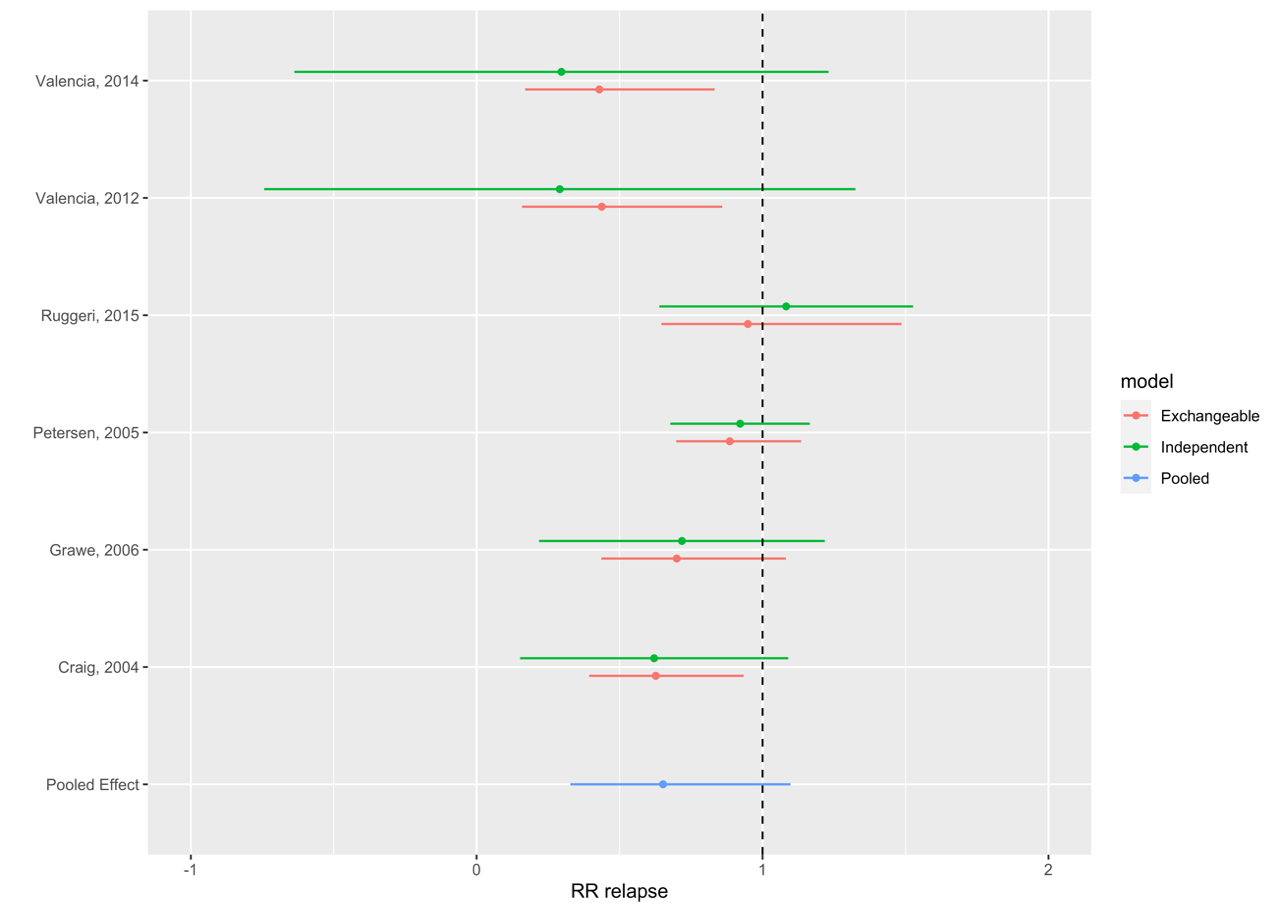 |
| --- |
| **Forest plot showing the results of a Bayesian random-effects meta-analysis of the effectiveness of EIP in terms of relapses. Each green point-and-range line represents the effect size and 95% credible interval (CrI) for each study assuming they are independent. The red point-and-range lines represent the effect sizes and 95% CrI assuming exchangeability. The blue point-and-range line is the pooled effect estimate with its 95% CrI. RR: risk ratio.** |

## Cost-effectiveness acceptability curves

In a cost-effectiveness acceptability curve (CEAC), the probability of an intervention being cost-effective is plotted against different thresholds of willingness to pay for a quality-adjusted life year (QALY) (Fenwick & Byford 2005). In the Figure below, the willingness to pay is expressed in Brazilian Real (R$). The WHO recommended threshold of 1 – 3 gross domestic per capita (GDPpc) is represented by the blue dashed lines.

Figure S4 Cost-effectiveness acceptability curve

| 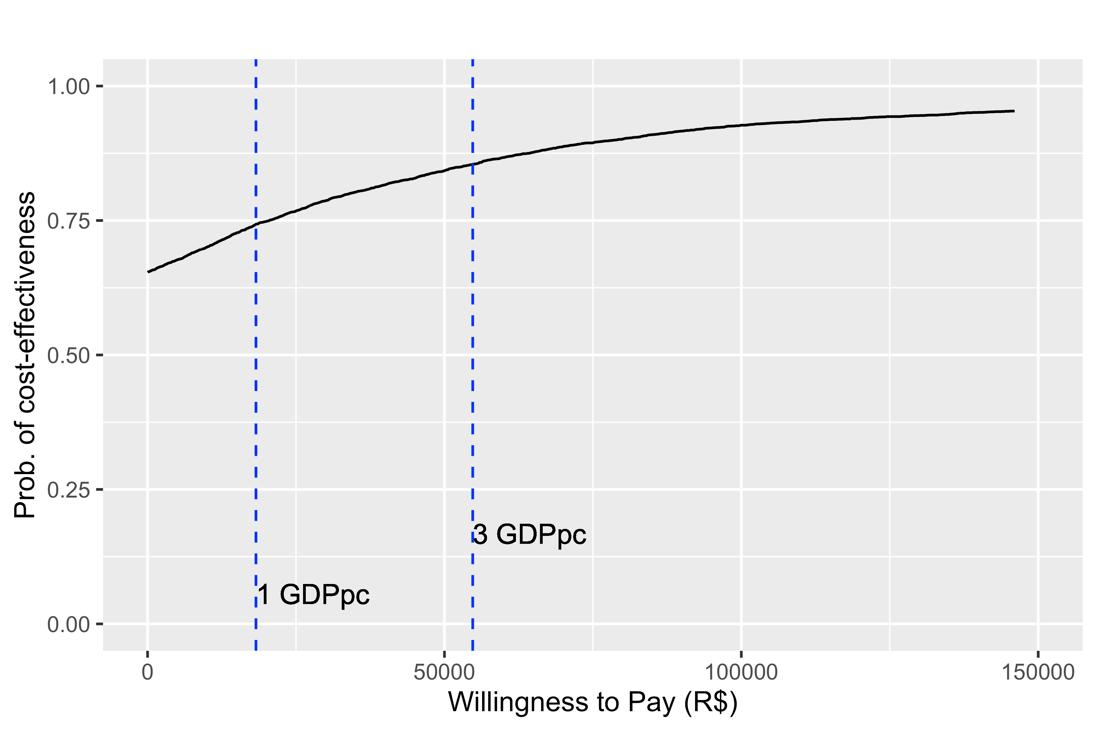 |
| --- |
| **CEAC: cost-effectiveness acceptability curve, EIP: Early Intervention in Psychosis, WTP: willingness to pay, GDPpc: gross domestic product per capita.** |

References

**Aceituno D, Pennington M, Iruretagoyena B, Prina AM and McCrone P** (2020) Health State Utility Values in Schizophrenia: A Systematic Review and Meta-Analysis. *Value in health: the journal of the International Society for Pharmacoeconomics and Outcomes Research* **23**, 1256–1267

**Alvarez-Jiménez M, Parker AG, Hetrick SE, McGorry PD and Gleeson JF** (2011) Preventing the second episode: a systematic review and meta-analysis of psychosocial and pharmacological trials in first-episode psychosis. *Schizophrenia bulletin* **37**, 619–630

**Becker P and Razzouk D** (2018) Cost of a community mental health service: a retrospective study on a psychosocial care center for alcohol and drug users in São Paulo. *Sao Paulo medical journal = Revista paulista de medicina* **136**, 433–441

**Buchanan RW, Panagides J, Zhao J, Phiri P, den Hollander W, Ha X, Kouassi A, Alphs L, Schooler N, Szegedi A and Cazorla P** (2012) Asenapine versus olanzapine in people with persistent negative symptoms of schizophrenia. *Journal of clinical psychopharmacology* **32**, 36–45

**Correll CU, Galling B, Pawar A, Krivko A, Bonetto C, Ruggeri M, Craig TJ, Nordentoft M, Srihari VH, Guloksuz S, Hui CLM, Chen EYH, Valencia M, Juarez F, Robinson DG, Schooler NR, Brunette MF, Mueser KT, Rosenheck RA, Marcy P, Addington J, Estroff SE, Robinson J, Penn D, Severe JB and Kane JM** (2018) Comparison of Early Intervention Services vs Treatment as Usual for Early-Phase Psychosis: A Systematic Review, Meta-analysis, and Meta-regression. *JAMA psychiatry*  **75**, 555–565

**Craig TKJ, Garety P, Power P, Rahaman N, Colbert S, Fornells-Ambrojo M and Dunn G** (2004) The Lambeth Early Onset (LEO) Team: randomised controlled trial of the effectiveness of specialised care for early psychosis. *BMJ*  **329**, 1067

**Davies LM, Barnes TRE, Jones PB, Lewis S, Gaughran F, Hayhurst K, Markwick A, Lloyd H and CUTLASS Team** (2008) A randomized controlled trial of the cost-utility of second-generation antipsychotics in people with psychosis and eligible for clozapine. *Value in health: the journal of the International Society for Pharmacoeconomics and Outcomes Research* **11**, 549–562

**Doyle R, Behan C, OʼKeeffe D, Masterson S, Kinsella A, Kelly A, Sheridan A, Keating D, Hynes C, Madigan K, Lawlor E and Clarke M** (2017) Clozapine Use in a Cohort of First-Episode Psychosis. *Journal of clinical psychopharmacology* **37**, 512–517

**Fenwick E and Byford S** (2005) A guide to cost-effectiveness acceptability curves. *The British journal of psychiatry: the journal of mental science* **187**, 106–108

**Galderisi S, Mucci A, Dollfus S, Nordentoft M, Falkai P, Kaiser S, Giordano GM, Vandevelde A, Nielsen MØ, Glenthøj LB, Sabé M, Pezzella P, Bitter I and Gaebel W** (2021) EPA Guidance on Assessment of Negative Symptoms in Schizophrenia. *European psychiatry: the journal of the Association of European Psychiatrists* 1–91

**Garcia-Gordillo MA, Collado-Mateo D, Olivares PR and Adsuar JC** (2018) Chilean population norms derived from the health-related quality of Life SF-6D. *The European journal of health economics: HEPAC: health economics in prevention and care* **19**, 675–686

**Grawe RW, Falloon IRH, Widen JH and Skogvoll E** (2006) Two years of continued early treatment for recent-onset schizophrenia: a randomised controlled study. *Acta psychiatrica Scandinavica* **114**, 328–336

**Howes OD, McCutcheon R, Agid O, de Bartolomeis A, van Beveren NJM, Birnbaum ML, Bloomfield MAP, Bressan RA, Buchanan RW, Carpenter WT, Castle DJ, Citrome L, Daskalakis ZJ, Davidson M, Drake RJ, Dursun S, Ebdrup BH, Elkis H, Falkai P, Fleischacker WW, Gadelha A, Gaughran F, Glenthøj BY, Graff-Guerrero A, Hallak JEC, Honer WG, Kennedy J, Kinon BJ, Lawrie SM, Lee J, Leweke FM, MacCabe JH, McNabb CB, Meltzer H, Möller H-J, Nakajima S, Pantelis C, Reis Marques T, Remington G, Rossell SL, Russell BR, Siu CO, Suzuki T, Sommer IE, Taylor D, Thomas N, Üçok A, Umbricht D, Walters JTR, Kane J and Correll CU** (2017) Treatment-Resistant Schizophrenia: Treatment Response and Resistance in Psychosis (TRRIP) Working Group Consensus Guidelines on Diagnosis and Terminology. *The American journal of psychiatry* **174**, 216–229

**Kaltenthaler E, Tappenden P, Paisley S and Squires H** (2011) NICE DSU Technical Support Document 13: Identifying and reviewing evidence to inform the conceptualisation and population of cost-effectiveness models. National Institute for Health and Care Excellence (NICE)

**Mena C, Gonzalez-Valderrama A, Iruretagoyena B, Undurraga J and Crossley NA** (2018) Early treatment resistance in a Latin-American cohort of patients with schizophrenia. *Schizophrenia research* **199**, 380–385

**Nishida A, Ando S, Yamasaki S, Koike S, Ichihashi K, Miyakoshi Y, Maekawa S, Nakamura T, Natsubori T, Ichikawa E, Ishigami H, Sato K, Matsunaga A, Smith J, French P, Harima H, Kishi Y, Fujita I, Kasai K and Okazaki Y** (2018) A randomized controlled trial of comprehensive early intervention care in patients with first-episode psychosis in Japan: 1.5-year outcomes from the J-CAP study. *Journal of psychiatric research* **102**, 136–141

**Oakley P, Kisely S, Baxter A, Harris M, Desoe J, Dziouba A and Siskind D** (2018) Increased mortality among people with schizophrenia and other non-affective psychotic disorders in the community: A systematic review and meta-analysis. *Journal of psychiatric research* **102**, 245–253

**Petersen L, Jeppesen P, Thorup A, Abel M-B, Øhlenschlaeger J, Christensen TØ, Krarup G, Jørgensen P and Nordentoft M** (2005) A randomised multicentre trial of integrated versus standard treatment for patients with a first episode of psychotic illness. *BMJ*  **331**, 602

**Randall JR, Vokey S, Loewen H, Martens PJ, Brownell M, Katz A, Nickel NC, Burland E and Chateau D** (2015) A Systematic Review of the Effect of Early Interventions for Psychosis on the Usage of Inpatient Services. *Schizophrenia bulletin* **41**, 1379–1386

**Razzouk D** (2019) Accommodation and Health Costs of Deinstitutionalized People with Mental Illness Living in Residential Services in Brazil. *PharmacoEconomics - open* **3**, 31–42

**Razzouk D, Kayo M, Sousa A, Gregorio G, Cogo-Moreira H, Cardoso AA and Mari J de J** (2015) The impact of antipsychotic polytherapy costs in the public health care in Sao Paulo, Brazil. *PloS one* **10**, e0124791

**Roberts M, Russell LB, Paltiel AD, Chambers M, McEwan P, Krahn M and ISPOR-SMDM Modeling Good Research Practices Task Force** (2012) Conceptualizing a model: a report of the ISPOR-SMDM Modeling Good Research Practices Task Force--2. *Value in health: the journal of the International Society for Pharmacoeconomics and Outcomes Research* **15**, 804–811

**Robinson S** (2008) Conceptual modelling for simulation Part I: definition and requirements. *The Journal of the Operational Research Society* **59**, 278–290

**Ruggeri M, Bonetto C, Lasalvia A, Fioritti A, de Girolamo G, Santonastaso P, Pileggi F, Neri G, Ghigi D, Giubilini F, Miceli M, Scarone S, Cocchi A, Torresani S, Faravelli C, Cremonese C, Scocco P, Leuci E, Mazzi F, Pratelli M, Bellini F, Tosato S, De Santi K, Bissoli S, Poli S, Ira E, Zoppei S, Rucci P, Bislenghi L, Patelli G, Cristofalo D, Meneghelli A and GET UP Group** (2015) Feasibility and Effectiveness of a Multi-Element Psychosocial Intervention for First-Episode Psychosis: Results From the Cluster-Randomized Controlled GET UP PIANO Trial in a Catchment Area of 10 Million Inhabitants. *Schizophrenia bulletin* **41**, 1192–1203

**Siskind D, McCartney L, Goldschlager R and Kisely S** (2016) Clozapine v. first- and second-generation antipsychotics in treatment-refractory schizophrenia: systematic review and meta-analysis. *The British journal of psychiatry: the journal of mental science* **209**, 385–392

**Siskind D, Siskind V and Kisely S** (2017) Clozapine Response Rates among People with Treatment-Resistant Schizophrenia: Data from a Systematic Review and Meta-Analysis. *Canadian journal of psychiatry. Revue canadienne de psychiatrie* **62**, 772–777

**Valencia M, Juarez F, Delgado M, Díaz A and Others** (2017) Early intervention to improve clinical and functional outcome in patients with first episode-psychosis. https://www.iconceptpress.com/book/mental-disorder/11000123/1305000979.pdf

**Valencia M, Juarez F and Ortega H** (2012) Integrated treatment to achieve functional recovery for first-episode psychosis. *Schizophrenia research and treatment* **2012**, 962371
